# Vein of Galen Aneurysmal Malformations: Updates on Technical Aspects and Functional Outcomes Post-Endovascular Treatment—A Systematic Review and Meta-Analysis

**DOI:** 10.3390/medicina60121948

**Published:** 2024-11-26

**Authors:** Talía Fuentes-Redondo, Pedro Navia-Álvarez, Luis-Alfonso Arráez-Aybar

**Affiliations:** 1 Department of Pediatrics, Complejo Hospitalario de Toledo, 45007 Toledo, Spain; tfuentesredondo@gmail.com; 2 Unit of Neuroradiology, La Paz University Hospital, 28046 Madrid, Spain; 3 Department of Anatomy and Embryology, Faculty of Medicine, Complutense University of Madrid, 28040 Madrid, Spain

**Keywords:** vein of Galen malformations, endovascular treatment, embolization, arteriovenous malformation, anatomical anomalies

## Abstract

*Background and Objectives*: Vein of Galen aneurysmal malformations (VGAMs) represent the most common vascular malformations of the brain at the pediatric age. Comprehension of its angioarchitecture and clinical features may influence their treatment options and functional outcomes. The aim of this review is to give an update of the anatomical and technical aspects of the management of VGAMs after endovascular treatment. *Materials and Methods*: We conducted a systematic review of original articles published between 1 January 2014 and 1 February 2024 in Pubmed, Web of Science (WOS), and Scopus databases following PRISMA guidelines. Variables such as age, sex, angioarchitecture of the malformation, clinical presentation, embolization technique, rate of occlusion, post-procedural complications, follow-up time, and mortality were collected. Random-effect meta-analysis of proportions was performed. *Results*: Fifteen studies on a total of 400 patients with VGAMs were collected. A total of 65.1% was male. The age at diagnosis was 12% prenatal, 35.5% neonates, 34.1% infants, 15.1% children, and 3.3% adults. Clinical presentation included 31.4% increased head size, 25.7% congestive heart failure, 12.9% neurological deficits, 10% seizures, 9.3% prominence of facial veins, and 8.9% developmental delay. A total of 339 patients underwent endovascular treatment (84.8%) with an average of 2.1 procedures per patient. The embolization technique was defined by transarterial access and glue material. Radiological occlusion was complete in 62.3% of the patients. The most frequent periprocedural incidents included hemorrhagic events (28.4%), embolization material migration (25.7%), and death (22%). The functional outcome was good in 68% of the surviving patients. The average follow-up time was 43.18 months. High heterogeneity was found in all outcomes but mortality rate. *Conclusions*: The angioarchitecture of VGAMs is significantly important when planning endovascular treatment and may have an influence on functional outcomes. More research into endovascular techniques and the risks of periprocedural complications must be performed. Indeed, a homogeneous protocol for the assessment of surviving VGAM patients during follow-up is necessary.

## 1. Introduction

Vein of Galen aneurysmal malformations (VGAMs) are the result of anomalous arteriovenous shunts between deep cerebral arteries and cerebral venous drainage through a primitive vessel named median prosencephalic vein of Markowski (MprosV), the embryonic precursor or the vein of Galen [1]. Although VGAMs are quite rare, they represent the most frequent pediatric vascular malformation with an estimated prevalence of 1 in 10,000–25,000 deliveries [2]. However, this prevalence might be underestimated because of the improvement in prenatal screening and the possibility of termination of pregnancy at the time of diagnosis [3]. VGAMs are reported to be equally frequent in both sexes and can be anatomically classified into two types: choroidal and mural [4,5]. There is also another type of malformation called vein of Galen aneurysmal dilatations (VGADs), but they are not the aim of this review.

The clinical onset of VGAMs may differ depending on age. In the neonatal group, the most frequent presentation is congestive heart failure, while in infants and children, neurological symptoms such as hydrocephalus, seizures, and macrocrania are more often reported. Adults and adolescents may present milder symptoms such as headaches and subarachnoid hemorrhages [2,4,6]. Without proper treatment, VGAMs usually have a fatal prognosis with a mortality ratio near 80–100% of the cases [7]. Advances in endovascular techniques have changed the course and prognosis of VGAMs from a high mortality finding to a potentially manageable condition. Transarterial embolization is the most utilized approach and might require multiple sessions to achieve complete occlusion of the VGAM, thus increasing the risk of procedural complications. Nonetheless, the main aim of endovascular treatment should be to achieve an acceptable level of occlusion to control cardiac failure and/or prevent the progression of neurological deficits with the lesser sequelae [4,6,8]. Clinical and radiological assessments are essential to evaluate these outcomes on a long-term basis although not many studies can provide extended follow-ups. 

The aim of this systematic review is to collect the latest findings about demographics, angioarchitecture, and clinical characteristics of VGAMs as well as to analyze the characteristics of the patients with VGAMs who underwent endovascular management including its complications and clinical and functional outcomes in order to envision further lines of research. 

## 2. Materials and Methods

Preferred Reporting Items for Systematic Reviews and Meta-Analyses (PRISMA) guidelines were applied [9]. This systematic review was included in the International Prospective Register of Systematic Reviews (PROSPERO) with the registration number CRD42024556459 URL: https://www.crd.york.ac.uk/prospero/display_record.php?ID=CRD42024556459 (accessed on 9 July 2024).

### 2.1. Information Sources and Search Strategy

We performed a systematic search in Pubmed, Web of Science (WOS), and Scopus databases searching for original articles published between 1 January 2014 and 1 February 2024 with the following terms and word variants for ‘vein of Galen malformation’, ‘vein of Galen aneurysmal malformation’, and ‘endovascular’. The search was limited to original articles in English or Spanish. Reference lists of relevant articles and Google Scholar sites were consulted to find additional records which might have been missed in the first search. We selected abstracts where basic demographic and angioarchitectural features of VGAMs were included as well as a description of the endovascular treatment performed and its complications and clinical and functional outcomes during follow-up. Editorials, narrative reviews, systematic reviews, and meta-analyses were excluded. 

### 2.2. Selection Process

The first two authors of the paper made the checklist by consensus before data extraction. Later, the same two authors performed the data extraction independently. Thus, we did not need to train any other researchers as the authors who made the checklist also performed the data extraction, assuring adequate consistency. In addition, any discrepancies between both authors during data extraction were resolved by the third author of this paper. First, title and abstract screening was performed in Endnote v20.6 software (PDFnet SKD©). Inclusion criteria were as follows: (1) any study (cohorts, case–control, and case series) that included patients undergoing endovascular treatment of VGAMs (with or without combination with other techniques such as microsurgery or stereotactic surgery); (2) any study containing a description of the endovascular technique (embolization material, approach route…), procedural complications and outcome, follow-up time, and mortality rate; (3) any study restricted to English or Spanish; (4) any study including ≥4 patients. Exclusion criteria were as follows: (1) no cases of aneurysmatic dilatations of vein of Galen (VGAD) and no cases of malformations resembling a VGAM; (2) no cases of spontaneous thrombosis of a VGAM; (3) no cases of vein of Galen varix; (4) no cases of patients who were not candidates for an endovascular treatment; (5) no narrative reviews, no systematic reviews and no meta-analysis.

### 2.3. Data Collection Process

Two authors extracted data independently following a defined checklist. Disagreements were resolved by consensus, and a third author was consulted if necessary. Data extraction included the following variables: (1) first author, (2) year of publication, (3) country, (4) study design, (5) study period, (6) no. of patients, (7) sex, (8) age at diagnosis, (9) VGAM angioarchitecture, (10) clinical presentation, (11) endovascular treatment specifications (no. of embolization sessions if possible, embolization material and endovascular route), (12) post-procedural outcome (technical issues and angiographic occlusion rate), (13) follow-up time (if available) and functional outcome, and (14) global mortality after endovascular treatment.

### 2.4. Risk of Bias Assessment

Risk of bias of the included studies was evaluated by using a modified Newcastle–Ottawa Scale (NOS) for cohort studies by two authors independently. NOS is used for quality assessment of nonrandomized studies included in systematic reviews and meta-analyses. According to this scale, three categories are evaluated for each study: selection, comparability, and outcome. When summing up, each study can score a maximum of 9 stars meaning low risk of bias when score is ≥7 stars.

### 2.5. Statistical Analysis

We performed a meta-analysis of proportions under a random effects model to report pooled data of the main outcomes observed. Heterogeneity among studies was evaluated with I^2^ statistic and a 95% confidence interval. I^2^ thresholds of <25% indicate low heterogeneity, 50% moderate heterogeneity, and 75% high heterogeneity. Statistical analysis was conducted with R software version 4.3.2 (R Core Team 2023, R Foundation for Statistical Computing, Vienna, Austria).

## 3. Results

### 3.1. Study Selection and Characteristics

**Figure 1:**
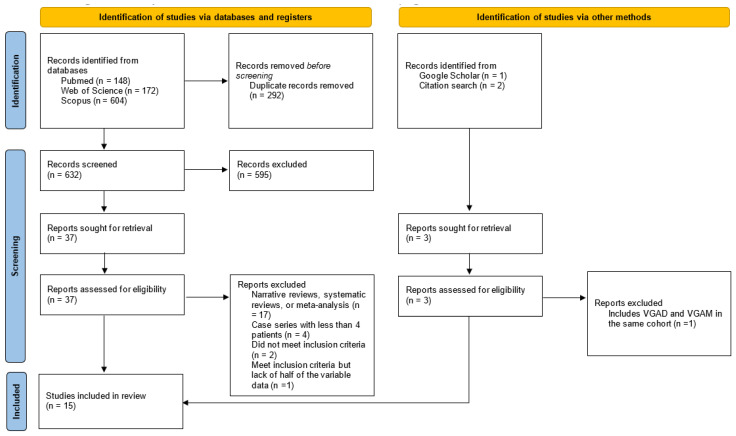
Study selection process. PRISMA flowchart.

A total of 924 abstracts were identified in all databases; 292 duplicates were removed by title. Of the 632 screened studies by title and abstract, 595 were excluded. Thirty-seven full-text studies were assessed for eligibility. Twenty-four articles were excluded due to the following reasons: 17 studies were narrative reviews, systematic reviews, or meta-analysis (exclusion criteria); 4 studies were case series with less than four patients (exclusion criteria), and 2 studies did not meet all inclusion criteria. The Malarbi et al. [10] study did meet the inclusion criteria, but it did not specify in full text more than half of the variables proposed for data extraction. When searching by other methods, we identified three possible studies that could also be assessed for inclusion and retrieved their full texts. After screening, the Giorgi et al. [11] study was excluded due to the inclusion of a VGAD mixed with a VGAM in the same case series for the variables proposed (exclusion criteria). Overall, we eventually included 15 studies in the systematic review. PRISMA flowchart for study selection is detailed in Figure 1, while the data extraction summary for each study is included in Table 1.

### 3.2. Risk of Bias in Studies

All articles included had a high risk of bias according to the NOS. A summary of the quality assessment is represented in Table 2. The main limitations of the selected studies were their retrospective design (intrinsic selection bias as the patients with VGAMs are recruited according to the treatment received and the possible outcomes have already occurred) and small sample size per study due to the low incidence of VGAMs which hindered the possibility of prospective studies or larger case series. There could be a publication bias as only high-volume or reference centers can report papers with more than four patients. There could also be a language bias. Functional outcome and mortality results might also be biased due to the short follow-up times and a lack of a standardized scale of functional evaluation among studies. Attrition bias can also be present due to a lack of data on certain variables collected within different studies due to its retrospective character.

### 3.3. Synthesis of the Results

A total of 400 cases of VGAMs were evaluated. Data about sex, age groups, angioarchitecture of the VGAMs, and clinical presentation at diagnosis were collected. Regarding to sex, 65.1% were male (252/387) and 34.9% were female (135/387). Cases were divided into five subgroups regarding age at diagnosis: 12% prenatal (36/299), 35.5% neonates (106/299), 34.1% infants (102/299), 15.1% children (45/299), and 3.3% adults (10/299). The weighted average of age among studies was 34.11 months (age range 0–57 years). Clinical presentation was available for 280 patients. The most frequent symptoms of VGAMs at diagnosis were as follows: 31.4% increased head size/hydrocephalus (88/280), 25.7% congestive heart failure (72/280), 12.9% neurological deficits (36/280), 10% seizures (28/280), 9.3% prominence of facial veins (26/280), 8.9% developmental delay (25/280), among others, such as headache, vision loss, epistaxis, feeding difficulties, failure to thrive, vomiting, hallucinations, and hemorrhage. A complete summary of these data is detailed in Table 3. 

Of the 400 patients with VGAMs, 339 underwent endovascular treatment (84.8%). A total of 380 embolization procedures were performed on the number of cases who underwent endovascular treatment with a weighted average among studies of 2.1 procedures per patient (range 1–7). The most frequently utilized embolization material was glue (215/278, 77.3%) followed by coils (37/278, 13.3%) or a combination of coils and glue (26/278, 9.4%). nBCA (n-butyl-cyanoacrylate) was used in 56.3% of the cases (121/215). The most frequent endovascular access (165/210, 78.6%) was transarterial followed by a combined transarterial plus transvenous approach (42/210, 20%), transvenous access alone being a rarity (3/210, 1.4%). The femoral artery was the first option in all but one study detailing endovascular access, which preferred carotid/vertebral artery. When exploring outcomes after endovascular treatment, variables such as occlusion rate, periprocedural complications (including death), functional outcome, follow-up time, and mortality were collected. Regarding radiological occlusion of the VGAM (also called occlusion rate or angiographic cure), complete occlusion was achieved in 62.3% of the patients (177/284), while partial occlusion rates were up to 37.7% (107/284). The rate of complications per embolization procedure was 22.9% (87/380), and complications were seen in 32.1% of the patients (109/339). The most common incidents relating to endovascular treatment were as follows: 28.4% hemorrhagic events (including intraventricular/subarachnoid hemorrhages) (31/109), 25.7% embolization material migration (28/109), 22% death (24/109), 10.1% ischemic events/infarction (11/109), among others, such as sinus thrombosis, femoral/iliac occlusion, hemodynamic instability, and vessel perforation. Functional outcome was classified as a good outcome in 68% (189/278) versus a poor outcome in 32% (89/278). Regarding follow-up time, these data could only be extracted from 8/15 studies with a weighted average of 43.18 months of follow-up, which ranged from 1 day to 16 years. The mortality rate after endovascular treatment was up to 7.1% of the patients (24/339). A complete summary of these raw proportions is detailed in Table 4, while pooled proportions of the main outcomes and their 95% confidence intervals are included in Table 5. Moderate to high heterogeneity (I^2^ values between 50 and 75% thresholds) was found in all variables but post-procedural mortality, which showed low heterogeneity (I^2^ 5.31%).

## 4. Discussion

### 4.1. VGAM Demographics, Angioarchitecture, and Clinical Presentation

Our systematic revision of about 400 patients showed many interesting outcomes. Regarding VGAM demographic data, this study showed a male predominance that was almost twice as frequent. This tendency was already observed in previous reports [3,4,24] although the literature often states an equal distribution of VGAMs between sexes [1,6,15,25]. The average age at diagnosis was over one year, but the age range was wide among the studies, with half of the patients being diagnosed during neonatal and infancy periods with a prospective increase in prenatal diagnosis. Diagnoses in children and adults are rarer [4,26]. Prenatal diagnoses of VGAMs has increased over the last years with improvements in prenatal screening and imaging techniques. Consequently, the necessity of studying signs of poor prognosis and termination of pregnancy protocols has been raised recently [19,25,27].

Age groups in this revision can be related to the clinical presentations most frequently found. In 1964, Gold et al. [28] reported the first classification of VGAM symptoms in each age group, and few changes were observed along this time. Neonates usually present with congestive heart failure, pulmonary hypertension, and organ disfunction, while infants and children usually present neurological signs such as macrocrania, hydrocephalus, prominence of facial veins, neurodevelopmental delay, and/or seizures with lesser grades of heart failure. Adults often show milder symptoms such as headaches, vomiting, or subarachnoid hemorrhages [4,7,29]. As half of the patients in this study belonged to the neonate and child age groups, it is feasible that the most common clinical onset data were related to the first-mentioned cardiac or neurologic signs.

Regarding VGAM angioarchitecture, the choroidal type was present in around 60% of the patients, matching the numbers reported in the previous literature [4,25,29,30]. The choroidal type is described as multiple bilateral shunts between the most anterior part of the MprosV and several arterial tributaries (mostly choroidal, pericallosal, subependymal, or thalamoperforating arteries). The mural type represented about 35% of the cases of this study, being described as a shunt between one or two arteries (mostly quadrigeminal or posterior choroidal arteries) directly into the lateral wall of the MprosV. Lately, some authors have also described a mixed type in which several high-flow fistulas end up at the lateral wall of the venous sac [4,8], but this type seems to be reported less. The deep venous drainage of the VGAM is also a matter of importance as there are different possibilities: a normal ‘Galenic’ drainage in which both internal cerebral veins terminate into the VGAM and/or an alternative drainage via the lateral mesencephalic vein into the superior petrosal sinus [20].

On the other hand, the angioarchitecture of the VGAM may also have an influence on clinical onset. Choroidal types tend to present sooner, normally at neonatal age, with severe congestive heart failure, while mural types tend to present later on in infancy with neurological deficits [2,4,6]. The high-volume shunt in choroidal VGAM and its complex anatomy with several arterial feeders involved lead to this early clinical presentation; thus, the choroidal type is usually related to a poorer prognosis [4,15]. 

### 4.2. Endovascular Treatment of VGAMs

Endovascular embolization is the first-line choice for definitive treatment of VGAMs since other techniques such as microsurgery, stereotactic surgery, and expectant management alone have increased morbidity and mortality rates. Khullar et al. [7] reported a mortality rate up to 84.6% in VGAMs treated with microsurgery. Nevertheless, the advances in endovascular techniques together with assessments by multidisciplinary medical experts such as obstetricians, pediatricians, interventional radiologists, and neurosurgeons have changed the course and prognosis of VGAMs [17,24]. 

Medical treatment of congestive heart failure is essential at neonatal onset to serve as a bridge of stabilization before a definitive procedure. Endovascular embolization can require multiple sessions to achieve complete occlusion in VGAMs. However, the main aim of the embolization of VGAMs is not to achieve complete occlusion of the malformation but an acceptable level of closure that allows for the control of cardiac failure and/or prevent the progression of neurological deficits [4,6,8,18,24]. 

Agarwal et al. [12] concluded that the rapid occlusion of the venous sac in one session should be avoided due to its higher risk of complications and mortality. In this systematic review, almost 85% of the patients included in the studies underwent endovascular treatment with an average ratio of 2.1 procedures per patient, similar to that reported by Lasjaunias et al. [31] and Brinjikji et al. [4], but a wider range of sessions (from 1 to 7) among the studies was also found. Hossman et al. [15] reported that choroidal types need more embolization procedures than mural types (2.6 vs. 1.4, respectively), a fact that Lasjaunias et al. [31], Berenstein et al. [6], and Habiba et al. [23] also stated. The high number of arterial tributaries in choroidal VGAMs makes it difficult to reduce the number of endovascular sessions. Besides, time between embolization procedures relays on vascular anatomy, patients’ age, and clinical condition. All these factors must always be re-evaluated before every endovascular procedure [6,15]. 

The age at the first endovascular treatment is also an important factor to consider. Due to the small size of the vascular tree, embolization in the neonatal group is challenging. Lecce et al. [14] concluded that embolization in neonates carried an increased risk of vascular injury, extravasation, and non-target embolization, which could contribute to the poorer outcomes in this age group. If possible, many authors recommend delaying the first endovascular session to around 4–6 months of age and always after an imaging update of the angioarchitecture of the VGAM and its venous drainage plus a thorough assessment of the clinical condition of the patient and the progression of the cardiac or neurological symptoms [2,6,15,32].

Sometimes, embolization during neonatal age cannot be avoided as controlling congestive cardiac failure becomes an urgent matter [32]. Lasjaunias et al. [31] proposed the Bicêtre neonatal evaluation score (BNES), a screening score that includes cardiac, cerebral, respiratory, hepatic, and renal parameters to assess the best moment for the beginning of definitive treatment during the neonatal period. A score between 8 and 12 points defines a clinically stable patient who needs a close clinical follow-up before performing embolization for VGAMs. This validated tool has demonstrated efficacy in the selection of candidates for endovascular treatment [4] although other authors have reported that BNES does not include imaging parameters, which are also very important in decision making, and it cannot be used beyond neonatal period [25].

Transarterial embolization (TAE) via the femoral artery was the most reported endovascular route to close VGAM. The TVE approach is not a first-line option to occlude VGAMs due to its increased risk of hemorrhagic events. A combination of TAE and TVE or even TVE alone can also be feasible only after several sessions of transarterial embolization when this access is no longer useful to achieve a higher occlusion rate of the malformation and residual shunts are still present [6,13,21]. The selection of the embolization material is indeed important. Glue is mostly preferred to coils alone as stated in our revision (77.3% vs. 13.3%). Acrylic glue materials allow for the use of smaller catheters which can be more accessible in the tortuous anatomy of the VGAMs. On the other hand, their main complication is the migration of the material to distal vessels. nBCA (n-butyl-cyanoacrylate) is preferred over Onyx (ethylene vinyl alcohol copolymer) in most procedures as it permanently sticks to the vessel wall. Berenstein et al. [6] proposed the use of nBCA as the first option but did not discard Onyx as a second-line after several nBCA embolizations. Other authors preferred the use of Onyx as it does not stick permanently to the vessel wall or to the catheter during the procedure and allowed a better control of the technique [7]. Orlov et al. [13] concluded that further studies must be performed in pediatric patients for better selection of the embolization material depending on the angioarchitecture of the VGAM. 

### 4.3. Clinical and Functional Outcomes After Endovascular Treatment

In a meta-analysis of pooled proportions, the complete occlusion rates in this systematic review (63.43%) were a little higher than those reported in the meta-analysis performed by Yan et al. [26] and Brinjikji et al. [4] (57% and 56%, respectively). As the aim of the endovascular treatment is more clinical than angiographical, these rates must be evaluated along with periprocedural complications, functional outcomes, and post-procedural mortality. The overall incidence of complications after endovascular treatment varies between 20 and 40% in the literature, so the one reported in our review (32.8%) was within those thresholds [4,25,26,29]. The most frequent incidents during or after an endovascular procedure were hemorrhagic events, death, migration of the embolization material, and ischemic events in similar proportions to previous studies [4,26]. Hemorrhagic and ischemic events could be prevented with a proper evaluation of the VGAM arterial tributaries and its venous drainage prior to the procedure [6,29]. Neonates suffer most from the complications, most probably due to the immaturity of the vascular tree and the complexity of the endovascular technique at this age [4,25]. Global mortality among the cohort of patients who underwent endovascular treatment was around 4.69%, which has significantly decreased over the last years, so endovascular treatment can be considered as an effective and secure technique [7,26,32].

Regarding functional outcome, a wide variability in evaluation scales and follow-up times was found among the studies included in this systematic review. The weighted average of the follow-up time was 43.18 months, but it ranged from 1 day to 16 years. Nevertheless, data about follow-up times were not available in all studies and information about the evaluation scales was not homogeneous. Evaluation scales such as the Jones scale [33], Bicêtre outcome score (BOS) [31], or King’s outcome scale for childhood injury (KOSCHI) are examples of the assessments utilized for evaluating good/poor outcomes. However, the items included in each of them are different, so good/poor outcome data might be strongly biased as a comparison among studies is hard to perform in these conditions and, moreover, only surviving patients were included for the functional outcome assessment. A detailed description of these facts is included in Table 6. Even so, a good outcome was found in 69.74% of the patients after endovascular treatment, similar to Yan et al. [26] and Brinjikji et al. [4] in their meta-analyses. Moreover, radiological (occlusion rate) and clinical/functional outcomes might differ in the same patient. Currently, there is increasing necessity for a homogeneous evaluation protocol for VGAM patients after treatment, including both short and long follow-up times.

### 4.4. Limitations of the Present Systematic Revision

Our systematic revision has limitations. The main weaknesses of the selected studies were the retrospective and case series designs (selection bias). This could also affect the availability of the data selected in each study due to its retrospective character (attrition bias). Lack of blinding and lack of randomization within the studies included are common to all meta-analyses. Another flaw is the small sample sizes per study as VGAMs have a low incidence, which makes it difficult to assess a cohort in a prospective study. There could also be a publication bias as only high-volume or reference centers can report papers with more than four patients, and it does not represent the experience of all providers performing VGAM embolizations. Because the level of success and post-procedural complications are operator-dependent, centers with a higher volume of patients and more experience might obtain better outcomes. In addition, some studies comprised several decades over which the treatment of VGAMs could have changed, misleading the outcomes. The language limitation to Spanish and English articles might also represent a publication bias. Also, the high mortality of the natural history of VGAMs and improvement in prenatal diagnosis can skew the outcomes as the most severe cases are not selected for endovascular treatment or they are offered a termination of pregnancy, which are not quantified in most studies (selection bias). Functional outcome and mortality results might also be biased due to a lack of follow-up time data of some of the studies included, lack of longer follow-up times of the studies, which included this item, and a lack of a standardized scale of functional evaluation among studies.

## 5. Conclusions

An exhaustive evaluation of the VGAM angioarchitecture is essential in planning endovascular treatment as it could significantly influence technical choices during embolization procedures. The wider our comprehension of VGAM anatomy, the greater the development of highly specific and less invasive treatment options. Studying the angioarchitecture of these complex malformations makes it possible to anticipate potential challenges and to use the most adequate techniques and materials for each case of VGAM, resulting in more precise and more effective endovascular interventions, with lesser procedural complications and better patient safety.

There is a necessity for a standardized protocol of assessment of VGAM patients before, during, and after treatment. This protocol should include clinical parameters together with anatomical/radiological features to refine the decision-making process and to be able to accurately foresee functional outcomes after treatment. The homogenization of VGAM assessments would unify management of these malformations among different centers and enable a better comparison of outcomes among studies.

In conclusion, a deep comprehension of the vascular anatomy of VGAMs is essential to optimize functional outcomes after treatment. Medical education and further research in this area will be the driving force for future advances in endovascular techniques and the improvement of these patients’ quality of life. Prioritizing anatomical knowledge both in clinical practice and research will have an impact on treatment strategies and long-term outcomes for patients with vascular malformations.

## Figures and Tables

**Table 1:** General characteristics of the studies included in the present systematic review.

First Author	Country	Study Design	Study Period	Sample Size (n)	Demographic Variables	Anatomical Variables	Endovascular Treatment Variables	Outcomes
Agarwal et al. [12]	India	Retrospective	1988–2015	36	S, AG, AA, AR	Ach, CF	No.P, No.E, EM, EA	OR, PC, FO, FT, M
Orlov et al. [13]	Russia	Retrospective	2011–2016	10	S, AG, AA, AR	Ach	No.P, No.E, EM, EA	OR, PC, M
Lecce et al. [14]	UK	Retrospective	2006–2016	34	S, AG, AA, AR	Ach, CF	No.P, No.E, EM, EA	OR, PC, FO, M
Hosmann et al. [15]	Austria	Retrospective	1975–2016	18	S, AG, AA, AR	Ach, CF	No.P, No.E, EM, EA	OR, PC, FO, FT, M
Gopalan et al. [16]	UK	Retrospective	2006–2016	31	S, MA, AR	Ach, CF	NoP. No.E, EM, EA	PC, FO, FT, M
Berenstein et al. [6]	USA	Retrospective	2004–2015	45	S, AG	Ach, CF	No.P, No.E, EM, EA	OR, PC, FO, M
Wagner et al. [17]	USA	Retrospective	2002–2018	18	S	Ach, CF	No.P, No.E, EM, EA	OR, PC, FO, FT, M
Sivasankar et al. [18]	India	Retrospective	1998–2012	26	S, AG, AR	Ach, CF	No.P, EM, EA	PC, FO, M
Taffin et al. [19]	France	Retrospective	2006–2009	52	S, AG, AA, AR	Ach	No.P, No.E, EM, EA	OR, PC, FO, M
Bhatia et al. [20]	Australia/ Canada	Retrospective	1999–2018	48	S, AG, AA, AR	Ach	No.P, No.E, EA	OR, PC, FO, FT, M
Brevis Nuñez et al. [3]	Germany	Prospective	2014–2015	30	S, AG	Ach, CF	No.P, No.E, EA	PC, M
Matsoukas et al. [21]	USA	Retrospective	2004–2021	10	MA, AR	Ach	No.P, No.E, EM, EA	OR, PC, FO, FT, M
Lauzier et al. [22]	USA	Retrospective	2002–2022	5	S, AG, AA, AR	CF	No.P, No.E, EM, EA	OR, PC, M
Habiba et al. [23]	Egypt	Retrospective	2015–2020	15	S, AG	Ach, CF	No.P, No.E, EM, EA	OR, PC, FO, FT, M
Nurimanov et al. [24]	Kazakhstan	Retrospective	2008–2022	22	S, AG, AR	Ach, CF	No.P, NoE, EM, EA	OR, PC, FO, FT, M

S—sex; AG—age group; AA—average age; AR—age range; Ach—angioarchitecture, CF—clinical features; No.P—no. of patients; No.E—no. of embolizations; EM—embolization material; EA—endovascular access; OR—occlusion rate; PC—periprocedural complications; FO—functional outcome; FT—follow-up time; M—mortality after endovascular treatment.

**Table 2:** Risk of bias assessment according to Newcastle–Ottawa Scale (NOS) for cohort studies for the studies included in the present systematic review.

First Author	Year of Publication	Selection	Comparability	Outcome
Agarwal et al. [12]	2016	٭٭	٭	٭٭٭
Orlov et al. [13]	2017	٭٭	٭	٭
Lecce et al. [14]	2018	٭	٭	٭٭
Hosmann et al. [15]	2018	٭٭	٭	٭٭٭
Gopalan et al. [16]	2018	٭٭	٭	٭٭
Berenstein et al. [6]	2018	٭٭	٭	٭٭
Wagner et al. [17]	2019	٭٭	٭	٭٭
Sivasankar et al. [18]	2019	٭٭	٭	٭٭
Taffin et al. [19]	2020	٭٭	٭	٭٭٭
Bhatia et al. [20]	2020	٭٭	٭	٭٭
Brevis Nuñez et al. [3]	2021	٭٭٭	٭	٭
Matsoukas et al. [21]	2022	٭٭	٭	٭٭
Lauzier et al. [22]	2023	٭٭	٭	٭
Habiba et al. [23]	2023	٭٭	٭	٭٭
Nurimanov et al. [24]	2023	٭٭	٭	٭٭

According to NOS, a study can be awarded a maximum of one star for each numbered item within the Selection and Outcome categories. A maximum of two stars can be given for Comparability.

**Table 3:** Summary of collected data about demographics and anatomical and clinical findings.

Variable		n (%)	Total (N)
Sex			387
	Male	252 (65.1%)	
	Female	135 (34.9%)	
Age group			299
	Prenatal diagnosis	36 (12%)	
	Neonates (<28 days)	106 (35.5%)	
	Infants (28 days–24 months)	102 (34.1%)	
	Children (2–18 years)	45 (15.1%)	
	Adults (>18 years)	10 (3.3%)	
Angioarchitecture			372
	Choroidal	229 (61.6%)	
	Mural	127 (34.1%)	
	Mixed	16 (4.3%)	
Clinical features at diagnosis			280
	Increased head size/ hydrocephalus	88 (31.4%)	
	Congestive heart failure	72 (25.7%)	
	Neurological deficits	36 (12.9%)	
	Seizures	28 (10%)	
	Prominence of facial veins	26 (9.3%)	
	Developmental delay	25 (8.9%)	
	Pulmonary hypertension	21 (7.5%)	
	“Organ disfunction”	18 (6.4%)	
	Headache	17 (6.1%)	
	Vision loss/proptosis	6 (2.1%)	
	Other	20 (7.1%)	

**Table 4:** Summary of collected data about endovascular procedure and outcomes observed.

Variable			n (%)	Total (N, %)
No. patients endovascular treatment				339 (84.8)
No. endovascular procedures				380
Embolization material	Glue			215 (77.3)
		nBCA	121 (56.3)	
		Onyx	17 (7.9)	
		Not specified	77 (35.8)	
	Coils			37 (13.3)
	Coils + glue			26 (9.4)
Endovascular access				210
	TAE		165 (78.6)	
	TVE		3 (1.4)	
	TAE + TVE		42 (20)	
Occlusion rate				284
	Complete		177 (62.3)	
	Partial		107 (37.7)	
Periprocedural complications				109 (32.1)
	Hemorrhagic events		31 (28.4)	
	Embolization material migration		28 (25.7)	
	Death		24 (22)	
	Ischemic events		11 (10.1)	
	Sinus thrombosis		5 (4.6)	
	Hemodynamic instability		5 (4.6)	
	Femoral/iliac occlusion		4 (3.7)	
	Vessel perforation		2 (1.8)	
	Other		5 (4.6)	
Functional outcome				278
	Good outcome		189 (68)	
	Poor outcome		89 (32)	
Mortality				24 (7.1)

TAE—transarterial embolization; TVE—transvenous embolization; nBCA—(n-butyl-cyanoacrylate).

**Table 5:** Pooled proportions for the outcomes collected in the present systematic review.

Outcomes		No. Studies (n)	Pooled Proportions, (95% CI)	I^2^, (95% CI)
Occlusion rate		13		83.35 (66.29–93.73)
	Complete		63.43 (51.20–75.66)	
	Partial		36.57 (24.34–48.80)	
Periprocedural complications		14	32.80 (20.25–45.36)	89.15 (78.98–95.37)
	Hemorrhagic events		35 (18.13–51.87)	86.07 (71.35–94.72)
	Death		24.62 (12.47–36.77)	72.63 (34.13–90.91)
	Embolization material migration		21.38 (9.08–33.69)	75.06 (47.73–90.79)
	Ischemic events		14.75 (4.93–24.57)	74.45 (55.29–95.99)
	Sinus thrombosis		5.7 (1.66–9.74)	0 (0–3.72)
	Femoral/Iliac occlusion		6.01 (1.78–10.24)	0 (null)
Functional outcome		12		66.01 (31.71–85.69)
	Good outcome		69.74 (61.05–78.44)	
	Poor outcome		30.26 (21.56–38.95)	
Mortality		15	4.69 (2.41–6.97)	5.31 (0–61.43)

**Table 6:** Summary of evaluation scales and follow-up times among the studies included in this systematic revision.

First Author	Good Outcome/ Poor Outcome (%)	Evaluation Scale	Average Follow-Up Time
Agarwal et al. [12]	74/26	NA	31 months
Lecce et al. [14]	50/50	Pediatric Stroke Outcome Measure (PSOM)	NA
Hosmann et al. [15]	54/46	Jones Scale	4.6 years
Gopalan et al. [16]	71/29	Recovery and Recurrence Questionnaire (RRQ)	23 months
Berenstein et al. [6]	70/30	Jones Scale	NA
Wagner et al. [17]	50/50	NA	38 months
Sivasankar et al. [18]	96/4	Bicêtre Outcome Score (BOS)	NA
Taffin et al. [19]	58/42	King’s Outcome Scale for Childhood Injury (KOSCHI)	1 phone assessment when patients were between 6 and 11 years of age
Bhatia et al. [20]	74/26	NA	44.7 months
Matsoukas et al. [21]	67/33	NA	17 months
Habiba et al. [23]	73/27	NA	23.47 months
Nurimanov et al. [24]	80/20	Jones Scale	58.87 months

NA—not available.

## Data Availability

Data are contained within the article. Dataset available on request from the authors.
